# A First-in-Class Dual Degrader of Bcl-2/Bcl-xL Reverses HIV Latency and Minimizes Ex Vivo Reservoirs from Patients

**DOI:** 10.3390/ijms26062772

**Published:** 2025-03-19

**Authors:** Lin-Chun Chang, Michael T. Yin, Gregory M. Laird, Kristen D. Ritter, Jayesh G. Shah, Asim K. Debnath

**Affiliations:** 1 Laboratory of Molecular Modeling and Drug Design, Lindsey F. Kimball Research Institute, New York Blood Center, New York, NY 10065, USA; 2 Department of Medicine, College of Physicians and Surgeons, Columbia University Irving Medical Center, New York, NY 10032, USA; mty4@cumc.columbia.edu (M.T.Y.);; 3 Accelevir Diagnostics, Baltimore, MD 21202, USA; greg.laird@accelevir.com (G.M.L.); kritter@accelevir.com (K.D.R.)

**Keywords:** HIV latency, viral reservoirs, Bcl-2/Bcl-xL, protein degrader, non-canonical, NF-kB

## Abstract

The persistence of latent HIV-1 proviruses in CD4^+^ T cells is a major obstacle to curing HIV. The “shock and kill” strategy involves reversing latency with latency-reversing agents (LRAs) and selectively inducing cell death in infected cells. However, current LRAs have shown limited efficacy in eliminating the ex vivo HIV reservoir and thus failed in clinical study. In this study, we repurposed PZ703b, a pro-apoptotic protein degrader initially developed for anti-leukemia therapy, to target HIV eradication. PZ703b induced the degradation of Bcl-2 and Bcl-xL, activating the non-canonical NF-kB pathway and caspases cascade, resulting in latency reversal and the selective apoptosis of infected cells. The treatment of ex vivo CD4^+^ T cells from ART-suppressed HIV-1 patients led to approximately a 50% reduction in the replication-competent reservoir. While this result does not reach the threshold required for a complete cure, it demonstrates the potential of a dual degrader of Bcl-2/Bcl-xL in reversing HIV latency and inducing selective cell death. Our study provides a proof-of-concept for using dual degraders of Bcl-2/Bcl-xL as a novel category of LRAs in therapeutic strategies aimed at reducing HIV reservoirs. This approach may pave the way for the further exploration of targeted interventions to eliminate the HIV-inducible reservoir.

## 1. Introduction

Combination antiretroviral therapy (cART) effectively suppresses HIV-1 replication but fails to achieve a cure due to the persistence of long-lived, resting memory CD4^+^ T cells that harbor latent and replication-competent HIV-1 DNA [1,2]. One potential curative strategy, known as “shock and kill” [3], involves the selective reactivation of HIV-1 gene expression using latency-reversing agents (LRAs), followed by the induction of cell death through virus-induced cytolysis or immune-mediated clearance of reactivated reservoirs [4,5,6]. Despite significant increases in plasma and cell-associated viral RNA following LRA treatment, clinical interventions have not yet resulted in substantial reductions in the latent reservoir [7,8,9]. Notably, some LRAs may inadvertently promote cell survival, counteracting attempts to eliminate infected cells [10]. This phenomenon might be attributed to the compromised activation of intrinsic cell death pathways, leading to inefficient or no elimination of cells expressing viral gene products.

Upon the reactivation of persistent HIV-1 infection by LRAs, viral proteins can interfere with apoptosis pathways, yielding varied outcomes, including activation, inhibition, or delay of cell death. For instance, the viral proteins Nef, Tat, and Vpr can impair apoptotic processes to promote cell survival and facilitate viral replication during the early stages of the viral life cycle. Conversely, in the late stages, the HIV envelope and Vpu proteins can promote apoptotic cell death [11]. Consequently, the balance between anti-apoptotic and pro-apoptotic cellular and viral proteins determines the fate of infected cells, influencing their survival or apoptosis [12]. Apoptosis is intrinsically regulated by members of the Bcl-2 family of proteins, which modulate apoptosis at the mitochondrial level. Key anti-apoptotic molecules in this family include Bcl-2 as well as BcL-2 homologs Bcl-xL, Bcl-W, A1, and Mcl-1 [13]. The inhibition of these proteins can lead to mitochondrial outer membrane permeabilization, resulting in cytochrome c release into the cytosol and the subsequent activation of the caspase cascade that executes apoptosis [12,14]. Evidence suggests that increased expression of the anti-apoptotic protein Bcl-2 can protect latently infected cells from apoptosis [15]. Thus, pharmacological approaches aimed at triggering intrinsic apoptotic pathways in reactivated HIV-infected reservoir cells present a promising strategy for viral reservoir elimination [16,17].

Pro-apoptotic compounds targeting Bcl-2 family proteins, extensively developed in cancer research [18], offer potential repurposing opportunities for HIV-1 reservoir elimination. For example, Navitoclax (ABT263), a dual inhibitor of Bcl-2 and Bcl-xL, and Venetoclax (ABT199), a selective Bcl-2 inhibitor, were initially designed as small molecule inhibitors targeting Bcl-2 family proteins in cancer therapeutics and have been investigated for their ability to induce cell death in HIV-infected cells [19,20,21]. However, the utility of ABT263 in clinical settings is limited by severe side effects, including on-target, dose-limiting thrombocytopenia [22,23]. As the only FDA-approved drug that targets the BCL-2 family protein [24] and is currently under clinical trials (NCT04886622), Venetoclax has demonstrated limited efficacy in directly reducing viral reservoirs in ex vivo experiments when administered as a single agent without the presence of cytotoxic T lymphocytes (CTLs) [20].

To mitigate the severe in vivo side effects associated with ABT263, Dr. Zhou’s group has developed a series of proteolysis-targeting chimeras (PROTACs) [25] exhibiting enhanced anti-leukemia efficacy while minimizing off-target toxicity [26,27,28]. PROTACs are bifunctional small-molecule degraders comprising two active ligands connected by a chemical linker. One ligand is designed to selectively bind to a protein of interest, while the other engages an E3 ubiquitin ligase. The recruitment of the E3 ligase to the target protein facilitates the formation of a ternary complex, which leads to ubiquitination and subsequent degradation of the target protein by cellular proteasomes [25,29]. By selecting cell or tissue-specific E3 ligases, PROTACs represent an innovative technology in precision medicine, enabling the targeted degradation of proteins in cancerous or infected cells with alternative protein expression [29,30]. Leveraging the characteristic low expression of Von Hippel–Lindau (VHL) E3 ligase in human platelets [27], Dr. Zhou’s group attached a ligand for the VHL E3 ligase to two different solvent-exposed rings of ABT263, leading to the discovery of two PROTACs, DT2216 and PZ703b, which exhibit selective cytotoxicity toward cancer cells while sparing platelets, thereby preventing thrombocytopenia [27,28]. Notably, both DT2216 and PZ703b have been shown to specifically degrade Bcl-xL, without concurrent Bcl-2 inhibitory activity, in lymphoblast cell lines derived from patients with lymphoblastic leukemia [27,28]. As DT2216 has recently entered clinical trials for the treatment of Bcl-xL-dependent relapsed or refractory peripheral T-cell lymphoma and cutaneous T-cell lymphoma (NCT04886622), the potential effects of DT2216 and PZ703b on the reduction in HIV-1 reservoirs remain to be explored.

Utilizing the advantages of protein degraders over traditional occupancy-based inhibitors allows for the circumvention of drug resistance arising from target protein overexpression or mutations, as protein degraders can degrade the entire protein [31,32]. Thus, this strategy has emerged as a promising antiviral approach to combat infectious diseases [33,34,35,36], although it remains underexplored in the context of HIV-1 infection. In this study, we aim to repurpose currently developed Bcl-2/Bcl-xL PROTACs to investigate the potential of protein-degrading modalities to target cellular proteins for degradation, ultimately achieving host-directed antiviral effects.

## 2. Results

### 2.1. Evaluation of BCL-2/BCL-XL Antagonists for Reactivating and Selective Killing of HIV from Latency

**Figure 1:**
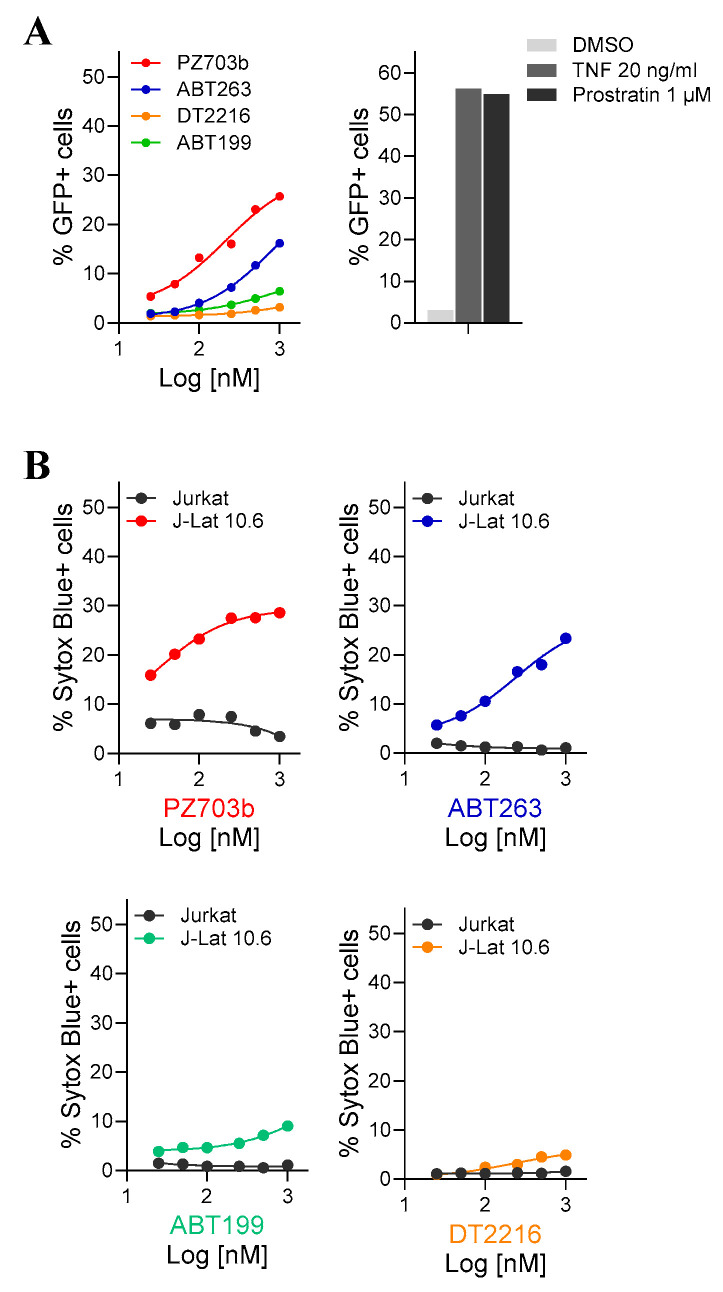
PZ703b exhibits potent HIV-1 reactivation and selective killing of J-Lat cells. Latently infected J-Lat 10.6 cells and their parental Jurkat cells were treated with serial concentrations of BCL-2/BCL-XL antagonists (PZ703b, ABT-263, ABT-199, and DT2216) for 48 h. TNF and Prostratin were used as positive controls, while DMSO served as the negative control. (**A**) HIV-1 reactivation was quantified by flow cytometry as the percentage of GFP-positive cells. (**B**) Cell death was assessed using SYTOX Blue, which is a nucleic acid dye that stains cells with compromised membranes. The percentage of SYTOX Blue-positive cells resulting from the drug’s anti-HIV effect was quantified by flow cytometry (Supplementary Figure S1) with the Jurkat cell data subtracted to isolate the effect in J-Lat cells.

To systematically evaluate the effectiveness of newly developed BCL-2/BCL-XL PROTACs and their predecessor small molecules in reactivating HIV from latency, we utilized J-Lat 10.6 cells, which is a well-established latently HIV-infected T cell line that expresses green fluorescent protein (GFP) upon HIV reactivation [37]. GFP expression correlates with viral reactivation in response to latency-reversing agents (LRAs). J-Lat 10.6 cells, along with their parental Jurkat cells, were treated with BCL-2/BCL-xL antagonists at varying concentrations for 24, 48, and 72 h. Dimethyl sulfoxide (DMSO) was used as the vehicle control, while TNF and Prostratin served as positive controls for viral reactivation. The parental Jurkat cells provided a background control. The level of GFP expression upon HIV reactivation was measured using flow cytometry, reporting the percentage of cells exhibiting increased fluorescence relative to the total cell count (Figure 1A). Compared to the approximately 55% HIV latency reversal observed in J-Lat 10.6 cells treated with TNF or Prostratin (Figure 1A, right), PZ703b induced a maximum of 25% reactivation at the highest concentration after 48 h of treatment with effects diminishing at lower doses (Figure 1A, left, red line). Treatment with ABT263 resulted in detectable GFP induction, though at lower levels than PZ703b at the same treatment period and concentration (Figure 1A, left, blue line). In contrast, DT2216 and ABT199 did not induce significant GFP expression throughout the treatment period (24, 48, and 72 h) (Figure 1A, left, orange and green lines, respectively). Obatoclax, a known BCL-2 antagonist, has previously demonstrated the ability to reactivate latent HIV-1 in J-Lat 10.6 cells at micromolar concentrations [38]. However, fluorescent signals were also detected in parental Jurakt cells treated with Obatoclax, indicating the spontaneous emission or excitation of signals, making it unsuitable for this model. Notably, PZ703b and ABT263, which act as dual antagonists of Bcl-2 and Bcl-xL in the Jurkat cell line (as demonstrated later in this report), achieved significant HIV reactivation at nanomolar concentrations, exhibiting superior potency compared to other screened BCL-2/BCL-xL antagonists.

To further investigate whether PZ703b and the other antagonists cause cytotoxicity in all treated cells or induce further cytopathic effects (CPE) in reactivated cells, we assessed cell death following the reversal of latent infection. The J-Lat 10.6 cell line contains a full-length integrated HIV genome with the Nef gene replaced by the GFP open reading frame and the Env gene suppressed by a frameshift mutation. This configuration permits the production of viral proteins associated with CPE [37]. Both Jurkat and J-Lat 10.6 cells were treated and monitored for cell death using SYTOX Blue, which is a dead cell stain that penetrates and intercalates nucleic acids only when the cell membrane is compromised. Neither TNF nor Prostratin treatment resulted in a substantial fraction of SYTOX Blue-positive cells in either cell line, reflecting their latency-reversing capabilities without significant cytotoxic effects. As drugs developed to killing lymphoma cells, PZ703b and ABT263 has significant toxicity to Jurkat cells when treat for 24, 48, and 72 h (Supplementary Figure S1). Additionally, after treatment for 48 and 72 h, both PZ703b and its predecessor, ABT263, exhibited significantly higher toxicity to J-Lat 10.6 cells (Figure 1B and Supplementary Figure S1), indicating that both compounds are highly effective in selective killing HIV-1 latently infected cells. DT2216 and ABT199 demonstrated slight cytotoxicity in J-Lat 10.6 cells compared to Jurkat cells (Figure 1B and Supplementary Figure S1). While well-known compounds such as prostratin and bryostatin can reverse HIV-1 latency without inducing significant CPE or cell death, additional strategies will be needed to eliminate reactivated latently infected cells. The results presented in Figure 1 demonstrate that PZ703b is the first compound shown to effectively reverse latent infection and reduce the latent reservoir alone.

### 2.2. PZ703b Reactivates Latent HIV Through the Non-Canonical NF-kB Signaling Pathway

**Figure 2:**
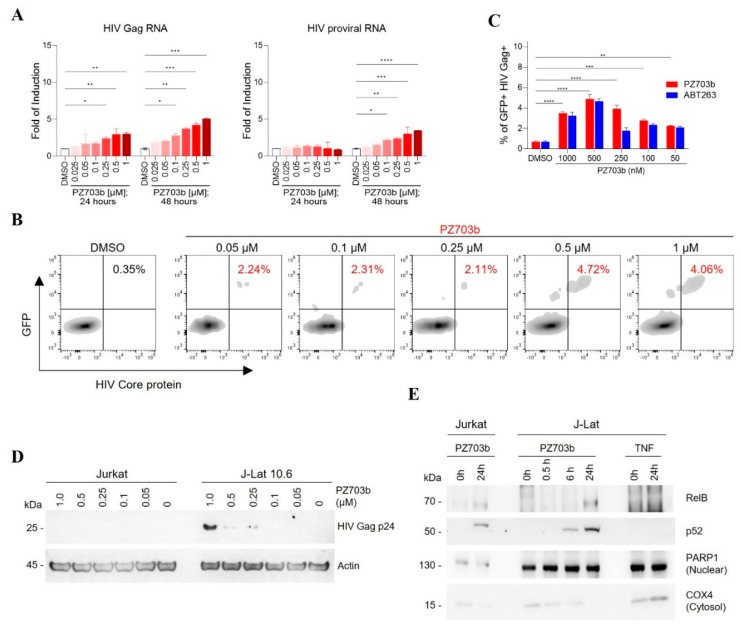
PZ703b reactivates HIV-1 latency in J-Lat cells. (**A**) RNA extracted from cells treated as indicated was analyzed for HIV Gag and proviral RNA content. The increased red color density indicates an increase in drug concentration. (**B**) J-Lat cells were treated, harvested, fixed, and permeabilized for intracellular staining of HIV-1 Gag proteins using an RD1 (Phycoerythrin)-conjugated KC57 antibody. The red-colored PZ703b and corresponding percentage indicate the effect of PZ703b treatment on the increase of GFP+ and Gag+ double-positive cells, compared to the dark-colored DMSO and its percentage. (**C**) Data from (**B**) and supplementary Figure S2B are presented as the mean ± SD with statistical significance (*p* ≤ 0.05) indicated by an arrow. (**D**) Western blot analysis detected the expression of HIV Gag p24 protein in response to increasing concentrations of PZ703b with Actin serving as the internal control. (**E**) Cells subjected to nuclear fractionation and treated as indicated were analyzed by Western blotting. COX4 and PARP1 were used as markers for cytoplasmic and nuclear proteins, respectively. Statistical significance was determined using two-way ANOVA: *, *p* < 0.05; **, *p* < 0.01; ***, *p* < 0.001; ****, *p* < 0.0001.

Our initial screening results indicate that dual antagonists of Bcl-2 and Bcl-xL can function as latency reversal agents (LRAs). To confirm the potency and efficacy of PZ703b and ABT263 in reactivating HIV-1 latency, we conducted experiments to measure the expression of HIV-1 transcripts and the HIV core protein Gag. We utilized real-time TaqMan quantitative PCR to assess HIV-1 transcript levels, while flow cytometry was employed to analyze HIV core protein expression before and after treatment with PZ703b or ABT263 with DMSO as a negative control and TNF as a positive control. In Figure 2A, we evaluated whether PZ703b can activate latent HIV gene expression, indicating full HIV reactivation and subsequent viral production. After administering PZ703b for 24 and 48 h, we observed a dose-dependent activation of HIV gag RNA transcription and latent HIV proviral RNA in J-Lat cells compared to the DMSO (negative control) and TNF (positive control) (Figure 2A, Supplementary Figure S2A). Moreover, both PZ703b and ABT263 significantly increased the frequency of cells expressing the HIV core protein Gag and the reporter GFP in a dose-dependent manner (Figure 2B,C, Supplementary Figure S2B). Western blot analysis further confirmed the induced expression of HIV Gag p24 in J-Lat cells following treatment with higher doses of PZ703b (Figure 2D). Collectively, these results demonstrate that PZ703b effectively activates the transcription of the latent HIV provirus in J-Lat cells, leading to the expression of both early gene products, such as Nef, and late gene products, such as Gag.

Bcl-2 and Bcl-xL have previously been implicated in the regulation of NF-kB pathway [39,40], which is a key target for several promising latency-reversing agents (LRAs) [41]. To investigate whether PZ703b activates the canonical NF-kB pathway, J-Lat cells were treated with DMSO or varying concentrations of PZ703b for 24 h, which was followed by intracellular staining for the phosphorylated p65 subunit of NF-kB and flow cytometry analysis. Treatment with PZ703b led to a significant decrease in phosphorylated p65, which typically translocate to the nucleus to initiate HIV transcription, compared to the DMSO mock control and the CD3/CD28-stimulated positive control (Supplementary Figure S2C). These results suggest that PZ703b reactivates viral latency independently of the canonical NF-kB pathway. Next, we examined the role of the non-canonical NF-kB (NF-kB2) pathway, which operates as a slow, persistent, and stimulus-selective mechanism that contrasts with and juxtapose canonical NF-kB signaling [42] and could be responsible for the PZ703b-dependent transcriptional activation of HIV. To assess this, we analyzed the nuclear translocation of NF-kB2 subunits following treatment with PZ703b, using PARP1 as a nuclear fraction marker and loading control, and COX4 as a cytosolic protein control (Figure 2E). Our findings demonstrated that the NF-kB2 subunits, p52 and RELB, translocated to the nucleus after 24 h of PZ703b treatment. In contrast, TNF, known to activate the canonical NF-kB pathway, did not induce the nuclear translocation of these subunits (Figure 2E). These data suggest that PZ703b functions as an atypical latency-reversing agent (LRA), reactivating latent HIV proviruses through activation of the non-canonical NF-kB2 pathway, which may help minimize off-target effects [42,43]. However, further experiments, including the knockdown or knockout of NF-κB2, are necessary to confirm the role of the non-canonical pathway in PZ703b-mediated HIV reactivation. Additionally, other signaling pathways may also contribute to this effect, and these will be explored in future studies.

### 2.3. Mechanisms of Selective Killing of HIV-Latently Infected Cells by PZ703b

**Figure 3:**
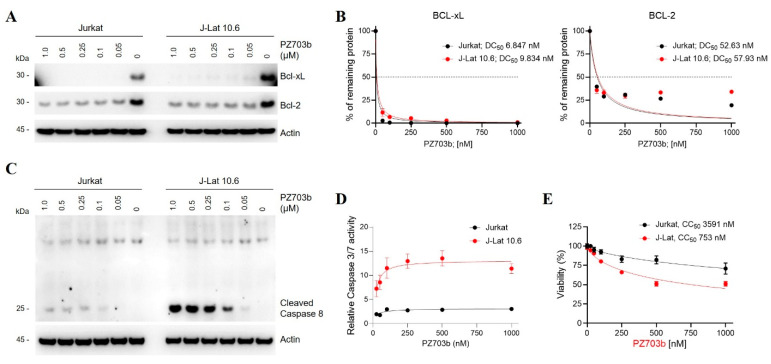
PZ703b degrades both Bcl-xL and Bcl-2 proteins and selectively induces apoptosis in HIV-latently infected cells. (**A**) Western blot analysis was performed to assess the protein levels of Bcl-xL, Bcl-2, and Actin after a 24 h treatment with the indicated concentrations of PZ703b. DC_50_ represents the drug concentration required for 50% degradation of the targeted proteins. (**B**) Actin was used as a loading control with untreated sample normalized to 100% for comparison. Protein levels in treated samples were referenced to Actin and normalized to calculate the reduction percentage. (**C**) Activation of the caspase cascade was assessed by detecting cleaved caspase 8. Cells were treated as described in (**A**), and lysates were analyzed by Western blotting using an anti-caspase 8 antibody. The 25 kDa band corresponds to cleaved caspase 8. (**D**) Enhancement of caspase 3/7 activity in PZ703b-treated J-Lat cells. Jurkat and J-Lat cells were treated with increasing concentrations of PZ703b for 24 h. Following treatment, cells were lysed and incubated with a caspase substrate (Promega), and luminescence was measured using a TECAN instrument. Two replicates were performed for each treatment group. (**E**) MTS assay in Jurkat and J-Lat cells incubated with increasing concentrations of PZ703b for 24 h. Data are presented as the mean ± s.d. from three replicate cultures in a representative experiment.

PZ703b and its sibling compound, DT2216, are newly developed PROTACs (proteolysis-targeting chimeras) that exhibit potent and safer anti-cancer properties compared to their warhead, ABT263 [27,28]. The target protein-binding moiety of PZ703b and DT2216 is derived from ABT263 [27,28], which is a dual inhibitor of Bcl-2 and Bcl-xL. Initially, it was hypothesized that both PZ703b and DT2216 could target and degrade Bcl-2 and Bcl-xL. However, previous studies have shown that both compounds specifically degrade Bcl-xL but not Bcl-2 in lymphoblast cell lines and lymphomas [27,28,44]. This selective degradation is likely due to differences in lysine preferences and the stability of the ternary complex formed for target protein ubiquitination and degradation [45]. In the present study, we first assessed the degradation efficiency and specificity of PZ703b and DT2216 on Bcl-xL and Bcl-2 in Jurkat and J-Lat cell lines. As previously reported, DT-2216 induced the degradation of Bcl-xL without affecting Bcl-2 in either cell line with a similar DC_50_ (half-maximal degradation concentration) to prior findings [27] (Supplementary Figure S3A,B). In contrast, PZ703b effectively degraded both Bcl-xL and Bcl-2 proteins after 24 h of treatment, even at a concentration of 50 nM, demonstrating superior efficacy compared to DT2216 in both cell lines (Figure 3A). The DC_50_ values for Bcl-xL and Bcl-2 degradation were approximately 10 nM and 50 nM, respectively, in both cell lines (Figure 3B). These results are consistent with previous findings indicating that DT2216 can only form a stable complex with Bcl-xL, while PZ703b can bind both proteins, as shown by NanoBRET assays [27,28]. Our data underscore the greater efficacy and specificity of PZ703b in degrading Bcl-xL and Bcl-2 in Jurkat T lymphocytes. While Jurkat cells are commonly used in leukemia research, the efficacy and specificity of PZ703b in modulating Bcl-2 and Bcl-xL levels may vary across different cell types and are independent of HIV infection.

Apoptosis is a regulated cell death process controlled by Bcl-2 and caspase family proteins, and it is initiated when Bcl-2 and Bcl-xL are lost, leading to mitochondrial outer membrane permeabilization and the release of cytochrome c into the cytosol. This event triggers the activation of initiator caspase 8 and subsequently the intrinsic apoptotic pathway. Given that PZ703b downregulates both Bcl-2 and Bcl-xL in Jurkat cells (Figure 3A,B), we hypothesized that PZ703b may activate intrinsic apoptosis. To investigate this, we measured active cleaved caspase 8 levels in PZ703b-treated Jurkat and J-Lat cells via Western blotting. As shown in Figure 3C, PZ703b significantly increased cleaved caspase 8 levels in J-Lat cells compared to Jurkat cells. Caspase 8 activation then leads to the cleavage of effector caspases 3 and 7 into their active forms. We further assessed whether PZ703b selectively enhances caspase 3 and 7 activity in latently HIV-1-infected cell lines. Jurkat and J-Lat cells treated with PZ703b were lysed, and caspases 3 and 7 activities were measured by incubating the lysates with a DEVD-containing substrate. Luminescence from PZ703b-treated cell lysates was normalized to DMSO-treated control. As a result of caspase 8 activation (Figure 3C), PZ703b treatment induced a five-fold increase in caspases 3 and 7 activity in J-Lat cells compared to Jurkat cells (Figure 3D). These findings highlight the selective activation of caspase cascades in J-Lat cells and demonstrate that the PZ703b-mediated killing of latently infected T cells is linked to enhanced apoptotic pathways in an HIV-dependent manner, counteracting anti-apoptotic molecules. To further evaluate the selective apoptotic effects of PZ703b on cell viability, we conducted MTS and trypan blue exclusion assays. PZ703b exhibited an approximately five-fold greater cytotoxicity against J-Lat cells (half-maximal cytotoxic concentration (CC_50_) of 0.75 µM) compared to Jurkat cells (CC_50_ of 3.5 µM) in both the MTS assay (Figure 3E) and the trypan blue exclusion assay (Supplementary Figure S3C). In contrast, DT-2216 showed no effect on the viability of either cell line in both assays (Supplementary Figure S3D), indicating that selective killing requires the concurrent degradation of both Bcl-2 and Bcl-xL in HIV-infected cells. Collectively, our results establish PZ703b as a novel dual degrader of Bcl-2 and Bcl-xL, which is effective at selectively killing HIV-latently infected cells.

### 2.4. PZ703b Does Not Lead to Global Activation of Primary T Cells

**Figure 4:**
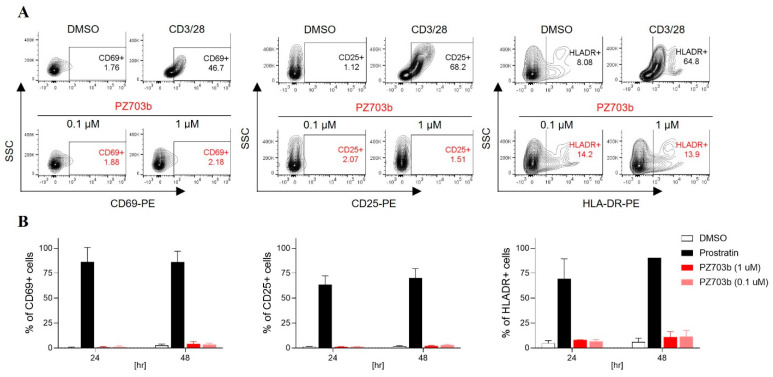
PZ703b does not induce global activation of T cells. Peripheral blood mononuclear cells (PBMCs) obtained from healthy donors were treated for three days (**A**) or 24 and 48 h (**B**) with 0.1% DMSO as a negative control, CD3/28 (**A**) or Prostratin (**B**) as positive controls, or PZ703b. Flow cytometry was used to assess the surface expression of activation markers (CD69, CD25, HLA-DR) on CD3^+^ T cells. The values represent the percentage of CD3^+^ cells expressing each activation marker, indicating the level of T cell activation.

A critical consideration for evaluating therapeutic candidates for HIV reservoir treatment is their ability to avoid global T-cell activation, which can lead to cytokine release and potential toxicity. In this study, we assessed the impact of PZ703b treatment on T cell activation. Human peripheral blood mononuclear cells (PBMCs) were isolated from two healthy donors and treated with varying concentrations of PZ703b for 72 h. As controls, we used CD3/28 beads and Prostratin, which is a protein kinase C (PKC) agonist known to activate both infected and uninfected bystander T cells—a major drawback of current latency reversal agents (LRAs) due to the risk of immune activation and potential collateral damage to healthy cells. We measured the surface expression of activation markers CD69, CD25, and HLA-DR on CD3-positive lymphocytes, including CD4^+^ and CD8^+^ T cells, from the treated PBMCs. The results shown in Figure 4A,B show that PZ703b treatment did not significantly increase the expression of activation markers at any concentration compared to the DMSO negative control. Importantly, PZ703b did not induce activation marker levels like those observed with CD3/28 or Prostratin treatment, which caused a significant increase in activation markers over the study period. The findings were consistent across both donors.

### 2.5. PZ703b Reverses Latency and Selectively Kills Reactivating Cells in the Central Memory T Cell (T_CM_) Model of HIV-1 Latency

During the HIV-1 life cycle, the expression of viral proteins can trigger cytopathic effects that lead to cell death. For example, the HIV envelope protein reduces BCL-2 levels, activating Bax and Bak, which form mitochondrial pores, leading to cytochrome C release and caspase cascades activation, ultimately driving apoptotic cell death [46,47]. Additionally, the HIV protease-generated Casp8p41 induces apoptosis, which can be counteracted by BCL-2 [48,49]. Furthermore, the HIV-1 accessory protein Vpu has been shown to suppress BCL-xL, which is significantly upregulated in individuals with HIV [50], thereby promoting apoptosis [51]. These findings suggest that reactivating latently infected HIV-1 cells could synergize with PZ703b to enhance cell death to reduce the HIV reservoirs’ size. To test this hypothesis, we utilized a central memory T cell (T_CM_) model of HIV latency, as described by Bosque’s group [52,53], which allowed representing the environment of peripheral blood mononuclear cells (PBMCs) isolated from HIV-1 patients undergoing various ART regimens. Naive CD4^+^ T cells were isolated from HIV-negative donor PBMCs via negative selection. The isolated CD4^+^ T cells were activated with anti-CD3/CD28 Dynabeads in the presence of anti-IL-4, anti-IL-12, and TGF-β before being infected with HIV-1 laboratory-adapted replication-competent viruses. After six days of infection, the cells were washed and cultured in a medium containing the HIV integrase inhibitor raltegravir (RLV) and the viral fusion inhibitor enfuvirtide (ENF) to drive the latency stage. After four days in culture with these antiretrovirals, latently infected central memory T cells were isolated for further study. HIV latency was confirmed using a cross-clade ultrasensitive real-time TaqMan PCR-based assay [54], which showed that latently infected cells contained integrated HIV-1 DNA with an average Cq value of 25, indicating approximately 300 to 3000 copies of HIV-1 latent proviruses [54] in both HIV-LAI and HIV-IIIB latently infected primary T cells, while uninfected T_CM_ cell were undetectable (Supplementary Figure S4A).

To prevent new rounds of infection while allowing the viral life cycle to complete, the two ARTs (RLV and ENF) were maintained, and we assessed whether PZ703b could activate latent HIV-1 and reduce the population of infected cells. Latently infected T_CM_ cells were treated with PZ703b for 72 h, and HIV reactivation was measured by intracellular staining of the HIV-1 Core protein with KC57 antibody, which was followed by flow cytometric analysis. DMSO served as a negative control, while anti-CD3/CD28 Dynabeads were used as a positive control for T cell receptor costimulation. The BCL-2 inhibitor ABT199, known to reduce the T_CM_ latency, was also included in our analysis [16]. HIV-1 Core protein-positive cells were gated from the total cell population to evaluate the effect of PZ703b on the reactivation. We found that neither PZ703b nor ABT199 reactivated HIV-1 Core expression in cells latently infected with HIV-LAI or HIV-IIIB compared to DMSO and anti-CD3/CD28 positive controls (Supplementary Figure S5A). While ABT199 induced significant toxicity in central memory T cells [20] (Supplementary Figure S5B), this effect appeared to be predominately in HIV Core protein-negative cells (Supplementary Figure S5C). These results suggest that PZ703b does not exhibit synergistic effects with viral factors when used in conjunction with integrase inhibitors and fusion inhibitors and therefore may not effectively reverse latency or selectively kill infected cells.

**Figure 5:**
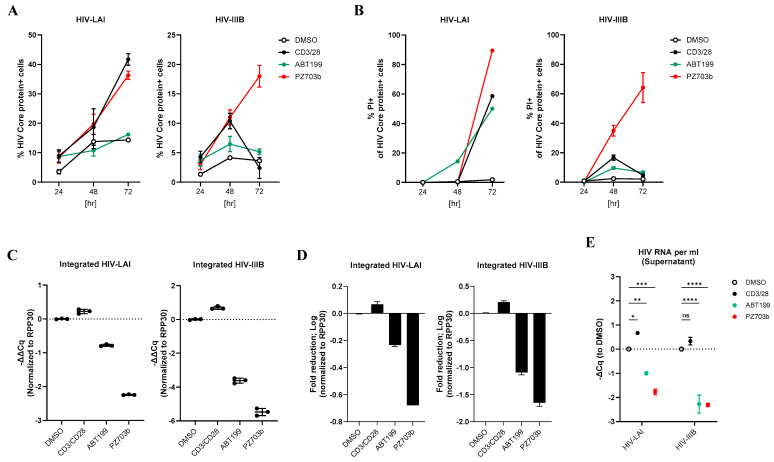
PZ703b reduces integrated HIV DNA in a T_CM_ model of latency through latency reversal and the selective killing of reactivating infected cells. Naive CD4^+^ T cells were isolated from the peripheral blood mononuclear cells (PBMCs) of healthy donors and established in a latent state by infection with HIV-LAI or HIV-IIIB/H9, or left as uninfected controls, as detailed in the Methods section. (**A**) The percentage of cells expressing the HIV core protein was assessed by intracellular staining with the KC57 antibody, which was followed by flow cytometry. (**B**) Apoptotic cell death among HIV core-expressing cells was determined using propidium iodide staining with apoptotic cells gated out from the HIV core-positive population. (**C**) Relative integrated HIV DNA were measured using nested TaqMan PCR after three days of treatment. (**D**) The fold reduction in integrated HIV DNA following three days of treatment was calculated relative to the DMSO control and transformed using a logarithmic scale. (**E**) Viral production in the supernatant was evaluated by measuring HIV RNA release in culture supernatants after three days of treatment in the presence of antiretroviral therapies (ARTs), RAL and DRV. Cq values from TaqMan RT-qPCR were normalized to DMSO control. Data are presented as mean ± SD from two biological replicates. Statistical significance was determined using two-way ANOVA: *, *p* < 0.05; **, *p* < 0.01; ***, *p* < 0.001; ****, *p* < 0.0001, ns indicated not significant.

In the HIV life cycle, the protease enzyme plays a crucial in the maturation of viral proteins [55,56]. Evidence suggests that fully processed viral proteins, such as Nef and Vpr, may protect virus-infected cells from apoptosis while simultaneously inducing the death of surrounding bystander cells [57,58]. Additionally, HIV protease activity within host cells can cause non-specific cleavage of various host proteins, including those involved in apoptosis [59,60,61,62]. Thus, viral protease can manipulate host mechanisms to enhance viral survival. Based on these observations, we hypothesized that treatment with a protease inhibitor could preserve critical host mechanisms, allowing PZ703b to exert its intended effects. To explore this hypothesis, we combined the HIV integrase inhibitor Raltegravir (RLV) with the viral protease inhibitor darunavir (DRV). This approach was used to evaluate the specific effects of PZ703b on the viral reservoir without the influence of mature viral proteins (Supplementary Figure S4B). After 72 h of treatment, PZ703b induced HIV Core protein expression in both HIV-LAI and HIV-IIIB latently infected T_CM_ cells compared to the DMSO control (Figure 5A). Additionally, PZ703b treatment resulted in a significant increase in cell death in the HIV Core-positive population (Figure 5B), indicating the selective killing of infected cells. In contrast, ABT199 did not reactivate HIV-1 Core expression in infected cells, which was consistent with previous studies showing a lack of latency reversal activity associated with ABT199 in HIV infection (Figure 5A). While ABT199 induced approximately 50% cell death in the HIV Core-positive population of HIV-LAI infected cells (Figure 5B), this effect was not selective, as ABT199 displayed cytotoxicity against the entire infected population (Supplementary Figure S6A, middle), which aligns with prior reports of ABT199’s non-specific toxicity to uninfected T_CM_ cells [20]. Notably, ABT199 did not induce selective cell death in HIV-1 IIIB latently infected T_CM_ cells (Figure 5B) and did not exhibit the non-specific toxicity seen in HIV-IIIB infection (Supplementary Figure S6A, right). Costimulation with CD3/28 enhanced CD69 surface expression, indicating robust T cell activation; however, neither PZ703b nor ABT199 significantly affected activation markers on infected or uninfected T_CM_ cells (Supplementary Figure S6B). PZ703b treatment also led to a reduction in the anti-apoptotic protein BCL-xL in both infected and uninfected T_CM_ cells (Supplementary Figure S6C), which was consistent with our previous observations in the J-Lat cell line (Figure 3). These results suggest that PZ703b can promote apoptotic cell death in cells with active HIV gene expression, though this effect is dependent on viral protease inhibition.

To further confirm that PZ703b selective kills latently infected cells, we extracted cell-associated DNAs to quantify integrated HIV-1 DNA and assess the impact of PZ703b on the reduction in the latent HIV-1 reservoir. Consistent with the observation that 60–80% of HIV Core-positive cells were apoptotic (Figure 5B), PZ703b treatment significantly reduced the amount of integrated HIV-1 DNA. This reduction, quantified as changes in ΔΔCq values, was normalized to the internal gene control RPP30 and the DMSO control in both virus-infected cell types (Figure 5C). This represented an approximate two-log decrease in the HIV-IIIB infected T_CM_ cells (Figure 5D). In contrast, ABT199 only modestly reduced the HIV reservoir size in the infected T_CM_ cells (Figure 5C,D) regardless of HIV Core protein expression levels (Figure 5A). Moreover, CD3/CD28 costimulation resulted in a slight increase in integrated viral DNA compared to DMSO controls (Figure 5C,D). Overall, these findings highlight that PZ703b has a more specific elimination effect in the T_CM_ model of HIV latency.

To investigate whether PZ703b influences viral dissemination to surrounding uninfected T cells, we collected supernatants for reverse transcriptase-PCR analysis to evaluate HIV RNA levels. While viral production was limited in the presence of ARTs, PZ703b further suppressed the release of viral particles, as evidenced by reduced HIV RNA levels in the supernatant (Figure 5E). This reduction in viral release aligns with the observed decreases in integrated HIV DNA (Figure 5C,D), suggesting that PZ703b has significant potential for restricting viral dissemination and reducing the size of the HIV reservoir ex vivo.

### 2.6. Ex Vivo Treatment with PZ703b Reduces HIV-1 Reservoir

The persistence of resting CD4^+^ T cells harboring integrated HIV-1 genomes presents a major barrier, as these cells can produce replication-competent viruses. In ongoing HIV cure-directed studies involving antiretroviral therapy (ART)-suppressed patients, the IPDA^®^ [63] has emerged as a promising method for quantifying replication-competent virus in reservoirs by distinguishing intact from defective proviruses, using a relatively small number of cells [64].

**Figure 6:**
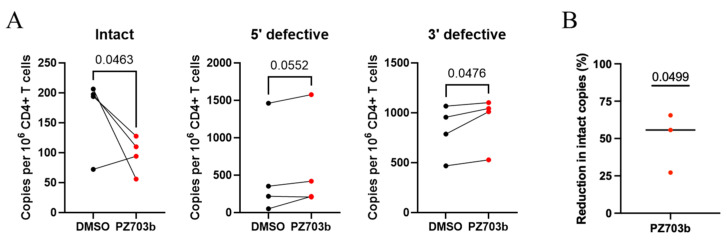
Ex vivo treatment with PZ703b reduces levels of intact HIV proviral DNA in samples from PWH on ART. (**A**) Frequencies of intact provirus^+^ CD4^+^ T cells (left), 5′-deleted provirus^+^ CD4^+^ T cells (middle), and 3′-deleted provirus^+^ CD4^+^ T cells (right) after treatment with PZ703b for three days. Data are reported as intact HIV-1 copies per million CD4^+^ T cells for each participant. Significance was determined by a one-tailed unpaired *t*-test. (**B**) The reduction in the frequency of intact provirus^+^ CD4^+^ T cells was calculated by dividing the average frequency of intact provirus^+^ CD4^+^ T cells after PZ703b treatment (left of (**A**)) by the frequency observed in the DMSO control. Black and red colored bullets indicate DMSO and PZ703b treatment, respectively. Data are shown as the percentage reduction (mean ± s.d.) from three out of four participants. Statistical significance was determined by one-sample t-tests against a theoretical mean of 0% reduction.

Building on our observations of PZ703b’s effectiveness in the T_CM_ model of HIV latency (Figure 5), we aimed to assess its potency in a preclinical setting. We tested whether PZ703b could drive the reduction in latent HIV ex vivo in CD4^+^ T cells isolated from four HIV-positive female adults (with or without HCV coinfection) who were on ART (Table I). The cells were treated with either DMSO or PZ703b in the presence of ART agents, including the integrase inhibitor RAL and the protease inhibitor DRV, for three days. Following treatment, the cells were analyzed using the IPDA assay to measure the frequency of intact provirus-positive cells. The IPDA quantifies intact HIV-1 proviruses as well as viral genomes with common defects, such as large internal deletions and hypermutation, which comprise the majority of reservoirs in people living with HIV [63,65,66]. While PZ703b has the potential to eliminate ex vivo reservoirs by inducing apoptotic death in infected cells, we did not isolate Annexin V-negative CD4^+^ T cells prior to the IPDA assay, which would have removed dying or dead cells [67,68]. Results showed that the frequency of intact provirus-positive CD4^+^ T cells was significantly lower in the PZ703b-treated group compared to the DMSO control group (Figure 6A, left). Three out of four participants experienced a reduction in intact viral copies per million cells with an average reduction of 50% in intact provirus levels observed in the PZ703b group relative to DMSO treatment (Figure 6B). No significant changes were observed in the frequencies of CD4^+^ T cells harboring 5′-defective proviruses among the four participants when comparing PZ703b treatment to DMSO (Figure 6A, middle). A significant increase in 3′ defective proviruses in the PZ703b-treated group compared to the control group was observed (Figure 6A, right). This could potentially be explained by the reduction in overall cell numbers in the treated group, as PZ703b exhibited an average toxicity of around 10% to CD4^+^ T cells (Supplementary Figure S7A). Most defective proviruses contain mutations in the tat and env genes, preventing a full reactivation of viral gene expression and viral protein production [65,66]. As a result, cells with defective HIV-1 may be less capable of triggering intrinsic apoptosis, further implicating viral reactivation in PZ703b-mediated reservoir reduction. Thus, the observed decrease in intact provirus^+^ CD4^+^ T cells likely reflects PZ703b-mediated cytotoxicity against HIV-1-infected cells containing replication-competent viral genomes. The post-recovery cell viability of ex vivo samples was modest (Supplementary Figure S7A), leading to an increased shearing index in both the DMSO and PZ703b groups (Supplementary Figure S7B). However, no significant difference between cell viability or DNA shearing was observed between PZ703b-treated cells and DMSO-treated cells, suggesting that these factors did not influence the assay outcomes. These findings provide direct evidence that ex vivo treatment with PZ703b eliminates latently infected CD4^+^ T cells people with HIV (PWH) on ART. Although the sample size is limited, these data are promising and warrant further investigation.

The relationship between HCV coinfection and HIV-1 reservoir size in cART-treated HIV patients remains complex and incompletely understood. Some studies suggest that HCV coinfection may increase the HIV-1 reservoir size, possibly due to chronic inflammation and immune activation [69]. However, other studies have found no such association [39]. Despite these mixed findings, ex vivo treatment with PZ703b showed limited effects on the reducing latent HIV in CD4^+^ T cells isolated from HCV-negative female adults (Supplementary Figure S7C). Given that HCV coinfection appears to sensitize latently infected CD4^+^ T cells to apoptosis in untreated HIV-positive patients [70], our preclinical observations underscore the importance of further investigating the interactions between HIV and HCV infections, and their impact on PZ703b-mediated reduction in the HIV reservoir.

## 3. Discussion

The “shock and kill” strategy aimed at eliminating the HIV-1 latent reservoir in CD4^+^ T cells relies on inducing latency reversal and viral reactivation, leading to either infected cell death or elimination by immune effector cells. In this study, we identified PZ703b, a novel protein degrader which like its parent compound ABT263, functions as a dual inhibitor of Bcl2 and Bcl-xL through protein degradation in Jurkat and J-Lat cell lines. We demonstrated that PZ703b and ABT263 effectively promote potent and specific caspase activation in a latently infected T cell line while sparing uninfected cells. Notably, ex vivo treatment with PZ703b in CD4^+^ T cells from people with HIV (PWH) eliminated up to 50% of cells containing intact proviruses, highlighting its potential as a promising therapeutic for reducing the HIV-1 reservoir. Given that ABT263 failed in phase II clinical trials due to on-target platelet toxicity, PZ703b offers a promising strategy for clinical development, demonstrating a favorable on-target profile with minimal platelet toxicity in the context of HIV reservoir reduction. Overall, our study positions PZ703b as the first-in-class protein degrader with potential applications in reducing reservoir size during chronic HIV infection.

To date, more than 160 compounds have been identified with latency-reversing activity; however, none of these latency-reversing agents (LRAs) have demonstrated a reduction in the size of the HIV reservoir or a delay in viral rebound in vivo [7,8,71]. Among these, pro-apoptotic molecules have been shown to activate NF-κB pathways and reactivate HIV-1 latency. SMAC mimetics, such as LCL-161, SBI-0637142, birinapant, and AZD5582, which target host anti-apoptotic factors like XIAP and cIAP1/BIRC2, serve as potent latency reversing agents (LRAs) by activating non-canonical NF-κB pathways [72,73]. This connection between apoptosis and latency reversal presents a promising strategy for reactivating the viral reservoir and promoting its selective elimination. In this study, PZ703b is established as the first pro-apoptotic protein degrader with demonstrated HIV reversal activity, specifically inducing selective toxicity toward HIV-infected cells and reducing the size of the ex vivo reservoir by 50%. Future studies could explore the potential synergistic effects of combining PZ703b with an SMAC mimetic.

In our study, we evaluated a range of pro-apoptotic molecules in the context of HIV infection, focusing on BCL-2/BCL-xL antagonists developed as cancer therapeutics and advanced to clinical trials. ABT-199 (Venetoclax), a selective BCL-2 inhibitor, has demonstrated efficacy in treating BCL-2-dependent hematological cancers [74] and is FDA-approved [24,75]. However, our results indicated that it only modestly reduced HIV-1 levels in latently infected J-Lat cell line and HIV-infected primary CD4^+^ T cells. Obatoclax (GX 15-070), a pan-BCL-2 family inhibitor, is capable of inducing apoptosis in cancer cells and reactivating HIV-1; however, we found that its effectiveness on latently infected cells varies depending on the methods employed [38]. In contrast, ABT-263, which inhibits both BCL-2 and BCL-xL, was found to be less potent than PZ703b in reversing viral latency and selectively killing J-Lat 10.6 cells. This underscores the necessity of simultaneously targeting both proteins for effective “kick-and-kill” strategies. PZ703b, a derivative of ABT-263, exhibited dual-targeting capability by effectively degrading both BCL-2 and BCL-xL proteins in Jurkat cells and Jurkat-derived HIV-latently infected cell lines. While PZ703b was confirmed as a selective BCL-xL degrader in lymphoblast cell lines [28], our findings indicate that differences between cell lines may influence its dual effects. Moreover, PZ703b exhibited enhanced activity at lower concentrations compared to ABT-263, highlighting the advantages of PROTACs over traditional small molecules [21]. It would be intriguing to further investigate the successors of PZ703b, specifically 753b and WH244, to evaluate their effectiveness in the same experimental framework as this study [26,45]. Overall, PZ703b presents a compelling therapeutic candidate for curative strategies against HIV-1 due to its specific targeting of HIV-infected cell death.

Shan et al. illustrated the disconnect between results obtained from primary-cell models and clinical efficacy [26,45], prompting researchers to focus on ex vivo CD4^+^ T cells from ART-treated donors [10]. In our study, we tested the ex vivo potency of PZ703b and demonstrated that it effectively reduces the ex vivo HIV reservoir from HIV/HCV coinfection patients, achieving nearly a 50% elimination of reservoir cells within three days of treatment. This result indicates that PZ703b’s efficacy is consistent across both in vitro cell lines and ex vivo reservoirs. Current studies indicate that while individual latency-reversing agents (LRAs) demonstrate significant activity in vitro, they lead to only minimal increases in HIV-1 RNA transcripts in ex vivo patient samples [68,76]. This modest reactivation may result in a partial decrease in the viral reservoir but does not achieve complete elimination. To further explore the ex vivo efficacy of PZ703b, future work will investigate its ability to reverse HIV latency in ex vivo settings, comparing its effects to those of PMA/I-treated cells, which are the standard for evaluating maximum reactivation [68,77,78,79]. Overall, this study establishes PZ703b as the first protein degrader identified as a latency-reversing agent (LRA), demonstrating potent efficacy in promoting HIV latency reversal and selectively inducing death in infected cells as a monotherapy.

## 4. Materials and Methods

### 4.1. Cell Line Culture

Jurkat and J-Lat full-length clone 10.6 cell lines [68,76] were obtained from the NIH AIDS Research and Reference Reagent Program [37] and maintained in RPMI-1640 GlutaMax (ThermoFisher, Bohemia, NY, USA) supplemented with 10% heat-inactivated fetal bovine serum and 1× MycoZap Plus-CL (Lonza Bioscience, Rockland, ME, USA).

### 4.2. Human Cell Isolation and Culture

Leukapheresis samples from healthy HIV-negative participants were also obtained from the New York Blood Center. Primary peripheral blood mononuclear cells (PBMCs) were isolated by density gradient centrifugation (STEMCELL Technologies, Cambridge, MA, USA). CD4^+^ T cells were purified using the EasySep Negative selection kit (STEMCELL Technologies, Cambridge, MA, USA). Cells were cultured in RPMI-1640 GlutaMax supplemented with 10% heat-inactivated FBS and 1× MycoZap (Lonza Bioscience, Rockland, ME, USA) without any cytokines or stimuli. Cryopreserved PBMCs were obtained from participants in a cohort study of postmenopausal women with HIV, which was led by Dr. Yin at Columbia University (IRB AAAF1644 and AAA2194 [80,81]). Inclusion criteria for this study included undetectable plasma HIV-1 RNA levels (<50 copies per mL). In total, samples from four women with HIV and Hepatitis C (HCV) coinfection and four women with HIV infection were included (see Table 1). The study protocol was approved by the Columbia University institutional review board, and all participants provided written informed consent.

### 4.3. In Vitro Cell Line Model for Screening BCL-2/BCL-XL Antagonists

Jurkat or J-Lat 10.6 cells were seeded in 24-well plates at a density of 1 million cells per mL per well. Cells were treated with either DMSO (0.1%) and TNF (20 ng/mL) or Prostratin (1 µM) as negative and positive controls, respectively, or with BCL-2/BCL-XL antagonists at serially diluted concentrations. After 24, 48, or 72 h of treatment, cells were harvested for GFP reporter measurement and assays.

### 4.4. Measurement of Supernatant HIV-RNA by TaqMan PCR

Viral particles were pelleted at centrifugation at 20,000 g for 2 h at 4 °C. The viral pellet was resuspended in 100 µL of residual medium, and RNAs were extracted using the QIAamp Viral RNA Kit (Qiagen, Germantown, MD, USA) following the manufacturer’s instructions. RNA concentrations were quantified, and 500 ng of viral RNA was mixed with 0.5 µM forward primer, 0.5 µM reverse primer, and 0.2 µM probe for qPCR, using the TaqMan™ Fast Virus 1-Step Master Mix (ThermoFisher, Bohemia, NY, USA). Samples were analyzed on a QuantiStudio 3 real-time TaqMan PCR system with default cycling parameters. Each run included tenfold serial dilutions of an RNA standard and negative control wells.

### 4.5. Protein Expression Analysis by Immunoblotting

Cells were treated with 0.1% DMSO (Sigma, Cat.# D8418) or serially diluted PZ703b (MedChemExpress, Monmouth Junction, NJ, USA) for 24 h. After treatment, cells were lysed and analyzed by Western blot. Cell lysates (20–50 µg) were separated on 412% NuPAGE Bis-Tris protein gels (Invitrogen, Carlsbad, CA, USA) and transferred to polyvinylidene difluoride (PVDF) membranes (BioRad, Hercules, CA, USA) overnight at 30 volts in 1× NuPAGE™ Transfer Buffer (Invitrogen, Carlsbad, CA, USA) containing 10% ethanol (Sigma-Aldrich, New York, NY, USA) and 0.05% SDS. Membranes were blocked in PBS with 5% bovine serum albumin (Sigma-Aldrich, New York, NY, USA) for 1 h at room temperature. After blocking, membranes were incubated with primary antibodies overnight at 4 °C, which was followed by incubation with HRP-conjugated secondary antibodies for 2 h at room temperature. Chemiluminescence was detected using Super Signal West Pico PLUS Chemiluminescent Substrate (ThermoFisher, Bohemia, NY, USA) and a BioRad ChemDoc system. The following antibodies were used: mouse anti-human caspase 8 (Cell Signaling Technology, Danvers, MA, USA), rabbit anti-human BCL-2 (Cell Signaling Technology, Danvers, MA, USA), rabbit anti-human BCL-xL (Cell Signaling Technology, Danvers, MA, USA), rabbit anti-actin (Cell Signaling Technology, Danvers, MA, USA), NF-κB2 p100/p52 (Cell Signaling Technology, Danvers, MA, USA), rabbit anti-human RelB (C1E4) (Cell Signaling Technology, Danvers, MA, USA), monoclonal anti-HIV p24 (NIH AIDS Reagent Program, New York, NY, USA), mouse anti-human PARP (Santa Cruz Biotechnology, Dallas, TX, USA), and mouse anti-human COX (Santa Cruz Biotechnology, Dallas, TX, USA).

### 4.6. Cytotoxicity MTS Assay

Jurkat or J-Lat 10.6 cells were seeded in 24-well plates at 1 million cells per mL. Cells were treated with DMSO or BCL-2/BCL-XL antagonists at serially diluted concentrations. After 24, 48, or 72 h, cell viability and cytotoxicity were assessed using the MTS assay (Promega, Madison, WI, USA), which measures cell proliferation and viability. The absorbance at 490 nm was recorded using a TECAN microplate reader, and data were analyzed using Magellan software (7.2 version).

### 4.7. Measurement of Caspase Activity

Jurkat or J-Lat 10.6 cells were treated with BCL-2/BCL-XL antagonists or DMSO for 24, 48, or 72 h. Caspase 3/7 activity was measured using the Caspase-Glo 3/7 Assay (Promega, Madison, WI, USA) according to the manufacturer’s instructions.

### 4.8. Flow Cytometry

After DMSO or PZ703b treatment for 24, 48, or 72 h, cells were fixed, permeabilized, and stained intracellularly with antibodies according to Thermo Fisher’s intracellular staining protocol. The following flow cytometry antibodies were used: PE-conjugated mouse anti-HIV core protein (Beckman Coulter, Brea, CA, USA), PE-conjugated mouse anti-human BCL-xL (Invitrogen, Carlsbad, CA, USA), and PE-conjugated rabbit anti-human NF-κB p65 (Ser536) (ThermoFisher, Bohemia, NY, USA). GFP-positive cells were determined using DMSO-treated J-Lat cells as a negative control. Cell death was assessed using Live/Dead Fixable Violet Dead Cell Stain (Invitrogen, Carlsbad, CA, USA) or Annexin V/PI kits (BD, San Jose, CA, USA) according to the manufacturer’s protocol. Flow cytometric analysis was performed on an Attune™ NxT Flow Cytometer (Invitrogen), and data were analyzed with FlowJo version 10 software (Tree Star).

### 4.9. Generated and Cultured Central Memory CD4^+^ Primary T Cells

Central memory CD4^+^ T cells were generated as previously described (52). PBMCs from HIV-negative donors were isolated by density gradient centrifugation STEMCELL Cat.# 18061). Resting CD4^+^ T cells were isolated using the Human Naïve T Cell Enrichment Kit (STEMCELL Technologies, Cambridge, MA, USA). The cells were cultured in RPMI-1640 GlutaMax medium supplemented with 10% heat-inactivated FBS, 1× Pen/Strep (ThermoFisher, Bohemia, NY, USA), and 1× MycoZap Plus-PR (Lonza Bioscience, Rockland, ME, USA). To activate and expand resting CD4^+^ T cells, cells were cultured with Dynabeads Human T-Activator CD3/CD28 (Invitrogen, Carlsbad, CA, USA), 2 mg/mL anti-human IL-12 (PeproTech), 1 mg/mL anti-human IL-4 (PeproTech), and 10 ng/mL transforming growth factor β1 (TGF-β1). After 3 days, Dynabeads were removed, and the cells were further cultured with 30 IU/mL IL-2 (Sigma-Aldrich, New York, NY, USA) for expansion.

### 4.10. Infection of Central Memory CD4^+^ T Cells to Establish Latency

On day 7 of the central memory T cell generation protocol, cells were infected with replication-competent HIV-1 viruses (HIV-IIIB or HIV-LAI) by spinoculation at 150 TCID50 for 2 h at 2000 × g and 37 °C. Infected cells were separated from the supernatant, suspended in fresh medium containing IL-2, and returned to the incubator. Non-infected control cells were cultured in parallel. On day 10, cells were washed and suspended at 1 million cells/mL in medium with IL-2 to promote crowd infection. On day 13, cells were treated with 1 µM Raltegravir and 0.5 µM darunavir or enfuvirtide to drive latency. On day 17, CD4^+^ T cells were isolated using magnetic positive selection and prepared for assays.

### 4.11. Analysis of Activation Markers in Human Primary T Cells

PBMCs were isolated from identified healthy donor blood. Human primary T cells were purified from human PBMC following the manufacturers’ manual (STEMCELL). The surface expression of CD69, CD25, and HLA-DR activation markers was analyzed by flow cytometry. Cells were cultured for 24, 48, and 72 h with the vehicle control DMSO or the following drugs: TNF, PZ703b, or ABT199 or costimulated with anti-CD3/CD28 antibody beads (Invitrogen). Each treatment contained one million cells. Post-treatment, fluorophore-conjugated antibodies targeting CD69, CD25, or CD69 were used to stain. Antibodies were incubated with cells at 4 degrees for 30 to 60 min and then washed with PBS at least twice before running flow cytometric analysis with ThermoFisher Attune NxT Flow Cytometer.

### 4.12. Measurement of Total Cell-Associated Integrated HIV Genome

DNA was extracted from at least one million cells using a Puergene DNA kit (Qiagen, Germantown, MD, USA) according to the manufacturer’s protocol and quantified using a NanoDrop spectrophotometer. Levels of total and integrated HIV DNA were measured as previously described with slight modification. Briefly, a first-step PCR mixture containing two forward primers specific for the human Alu elements and a reverse primer specific for the HIV Gag gene was followed by a nested real-time TaqMan PCR with a pair of forward and reverse primers and a probe able to amplify both total and integrated HIV genome. TaqMan Quantitative PCR was performed using HIV primers and probers to cross-clade detect for HIV integrated and total DNA and a primer–probe set for RPP30 (RNase P) as an internal control. PCR reaction mixtures were prepared by mixing 500 ng of purified DNA, 1000 nM of primers, and 250 nM of probes with TaqMan™ Gene Expression Master 2× Mix (Applied BioSystem, Foster City, CA, USA). The PCR reaction was then conducted in a QuantiStudio 3 real-time PCR system with the default condition setting. The Cq values of HIV DNA were normalized to the Cq values of RPP30. Integrated or total HIV DNA was calculated and expressed in one million CD4^+^ T cells by the standard number of HIV DNA copies from the serial dilution of ACH2 DNA standard controls.

### 4.13. Ex Vivo Elimination of Latent Reservoir

CD4^+^ T cells were isolated from cryopreserved PBMCs of participants on suppressive ARTs (Table 1) and plated in cultured in RPMI-1640 GlutaMax supplemented with 10% heat-inactivated fetal bovine serum and 1× MycoZap (Lonza Bioscience, Rockland, ME, USA) with PZ703b and ARTs (10 M DRV and 10 M RAL) and incubated at 37 °C for 72 h. The CD4^+^ T cells were then washed with complete RPMI medium to remove the treatment and subjected to IPDA assay.

### 4.14. IPDA^®^ Assay

An in-depth description of the IPDA rationale and procedure is available in Bruner et al. [63]. In this study, the IPDA was performed by Accelevir Diagnostics under company standard operating procedures. Cryopreserved PBMCs from each participant were thawed, and total CD4^+^ T cells were obtained via immunomagnetic selection (EasySep Human CD4^+^ T cell Enrichment Kit, Stemcell Technologies, Cambridge, MA, USA) with cell count, viability, and purity assessed both before and after selection. An average of two million untouched CD4^+^ T cells were recovered for each sample and equally divided for DMSO or PZ703b treatment. Genomic DNA was isolated using the QIAamp DNA Mini Kit (Qiagen, Germantown, MD, USA). DNA concentrations were determined by fluorometry (Qubit dsDNA BR Assay Kit, Thermo Fisher, Bohemia, NY, USA), and DNA quality was determined by ultraviolet-visible (UV/VIS) spectrophotometry (QIAxpert, Qiagen, Germantown, MD, USA). Genomic DNA was then analyzed by IPDA^®^. Up to 700 ng of genomic DNA was analyzed for each proviral discrimination reaction, while copy reference/shearing reactions used diluted (1:100) samples with a maximum of 7 ng of genomic DNA analyzed per reaction. Samples were processed and analyzed in batches by blinded operators. The copy reference/shearing component reaction serves two purposes in the IPDA. First, this component assay calculates the number of input diploid genome equivalents for the IPDA by targeting the human RPP30 gene. Second, this component assay allows us to quantify and correct the impact of DNA shearing on intact proviral genome quantification. An in-depth description of this component of the IPDA is provided by Bruner et al. (63).

### 4.15. Statistical Methods

Statistical analyses were performed using GraphPad PRISM version 10 software. The specific statistical methods used for each experiment are detailed in figure legends. A *p*-value of ≤0.05 was considered statistically significant.

## Figures and Tables

**Table 1:** Clinical features of the four participants with HIV whose peripheral blood mononuclear cells were used in the ex vivo study.

ID	Age	Sex	HCV	Height (cm)	Weight (kg)	HIV Regimen	CD4 T Cells	Viral Load
1	49	Female	+	167.9	105	Abacavir, lamivudine, darunavir/ritonavir	953	<50
2	57	Female	+	157.5	79.4	Tenofovir disoproxil fumarate, nelfinavir	794	<50
3	65	Female	+	147.6	56.5	Tenofovir disoproxil fumarate, lamivudine, lopinavir/ritonavir saquinavir	1144	<50
4	57	Female	+	157.5	79.4	Tenofovir, lamivudine, nelfinavir	794	<50
5	47	Female	-	171.7	87.5	darunavir/ritonavir	750	<50
6	48	Female	-	168.15	75	darunavir/ritonavir	731	<50
7	64	Female	-	152.4	52.6	lopinavir/ritonavir	746	<50

## Data Availability

All data and materials presented in the study will be available to researchers for the purpose of reproducing or extending the analysis upon acceptance of the study for publication.

## Supplementary Materials

The following supporting information can be downloaded at https://www.mdpi.com/article/10.3390/ijms26062772/s1.
