# Supplementary material for: A First-in-Class Dual Degrader of Bcl-2/Bcl-xL Reverses HIV Latency and Minimizes Ex Vivo Reservoirs from Patients
Source: Int J Mol Sci. 2025 Mar 19;26(6):2772. doi: 10.3390/ijms26062772 (PMC11942780; doi:10.3390/ijms26062772)
Supplement: Supplementary file 1 [file ijms-26-02772-s001.zip › ijms-3453482-supplementary.pdf]

Supplementary

A First-in-Class Dual Degrader of Bcl-2/Bcl-xL Reverses HIV Latency and Minimizes Ex Vivo Reservoirs from Patients

Lin-Chun Chang [1,*], Michael T. Yin [2], Gregory M. Laird [3], Kristen D. Ritter [3], Jayesh G. Shah [2] and Asim K. Debnath [1,*]

1 Laboratory of Molecular Modeling and Drug Design, Lindsey F. Kimball Research Institute, New York Blood Center, New York, New York, USA.
2 Department of Medicine, College of Physicians and Surgeons, Columbia University Irving Medical Center, New York, New York, USA.
3 Accelevir Diagnostics, Baltimore, Maryland, USA.
* Correspondence: Lin-Chun Chang (LChang@nybc.org); Asim K. Debnath (adebnath@nybc.org); Laboratory of Molecular Modeling and Drug Design, Lindsey F. Kimball Research Institute, New York Blood Center, New York, New York

One Sentence Summary: PZ703b is a groundbreaking protein degrader with LRA activity that uniquely shocks and eliminates HIV latently infected cells while also being the first LRA monotherapy to demonstrate significant ex vivo reduction of HIV reservoirs, highlighting its potential as a transformative therapeutic strategy against HIV.

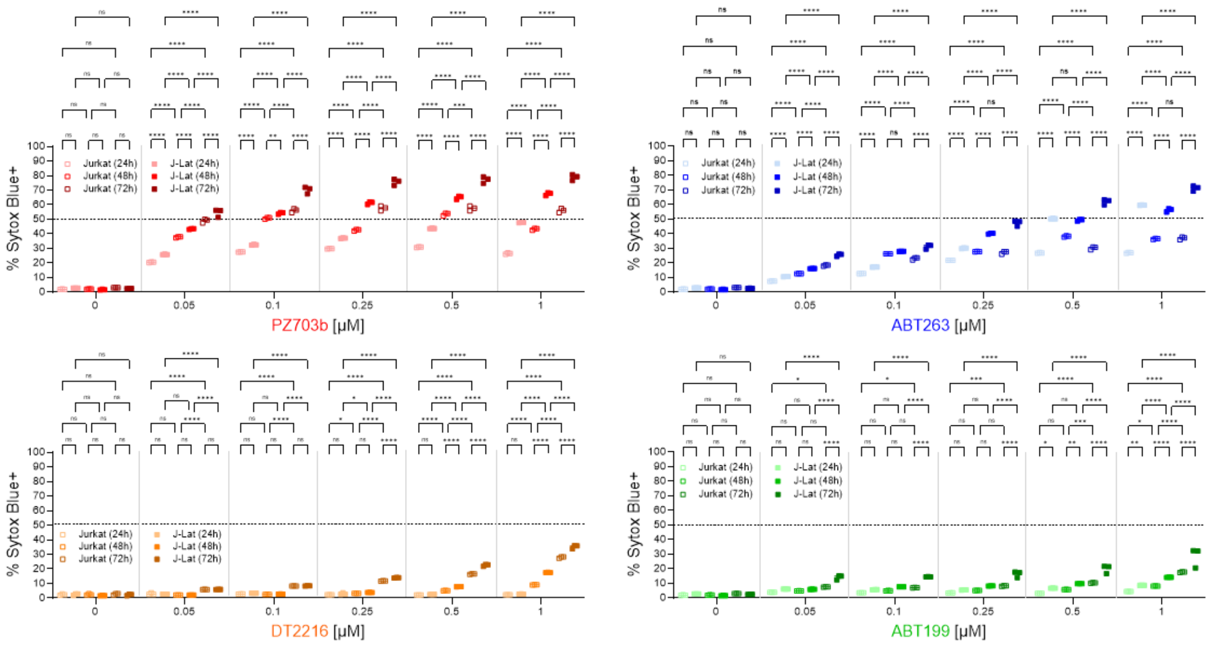

Supplementary Figure S1. Screening of BCL2/BCL-XL antagonists reveals that PZ703b and ABT263 selectively increase cell death in HIV-1 latently infected cells in a dose-dependent manner. Jurkat and HIV-latently infected J-Lat 10.6 cells were treated with serial concentrations of BCL2/BCL-XL antagonists for 24, 48, and 72 hours. Cell death was monitored by SYTOX Blue staining, which identifies permeabilized (dying) cells, and was quantified by flow cytometry as the percentage of SYTOX Blue-positive cells from total cells.

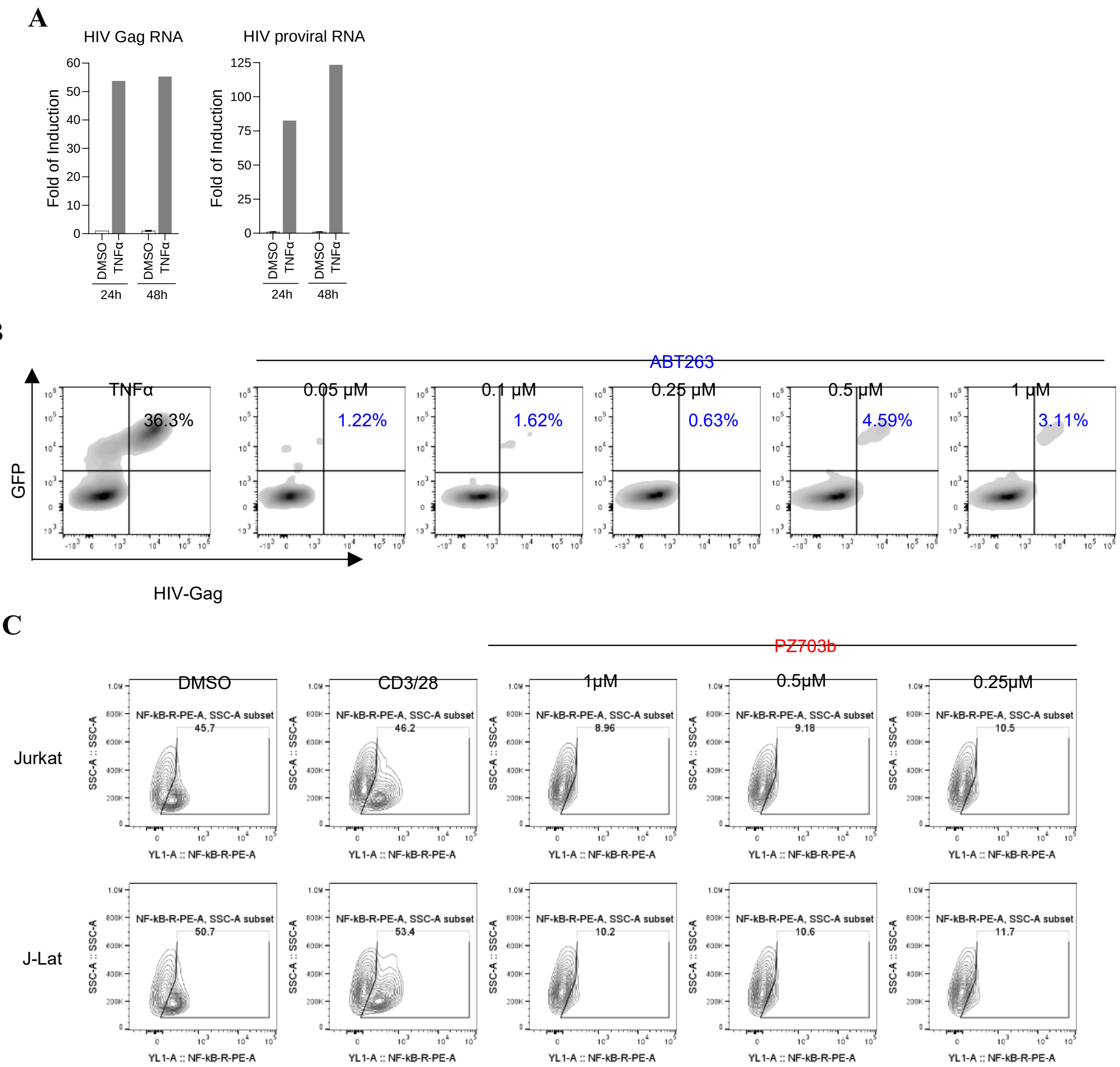

Supplementary Figure S2. ABT263 Reactivates HIV Latency in J-Lat Cells, and PZ703b Represses Canonical NF-kB Pathway Signals (A) RNA isolated from cells treated as indicated was analyzed for HIV Gag and proviral RNA content. **(B)** Intracellular staining of HIV-1 Gag proteins demonstrates the effectiveness of PZ703b in reversing latent viral infection. Cells were treated, harvested, fixed, and permeabilized for intracellular staining of HIV-1 Gag proteins using an RD1 (Phycoerythrin)-conjugated KC57 antibody. **(C)** PZ703b treatment resulted in decreased NF-kB activity compared to DMSO and CD3/CD28 stimulation controls, which is consistent with its effects on T cell activation.

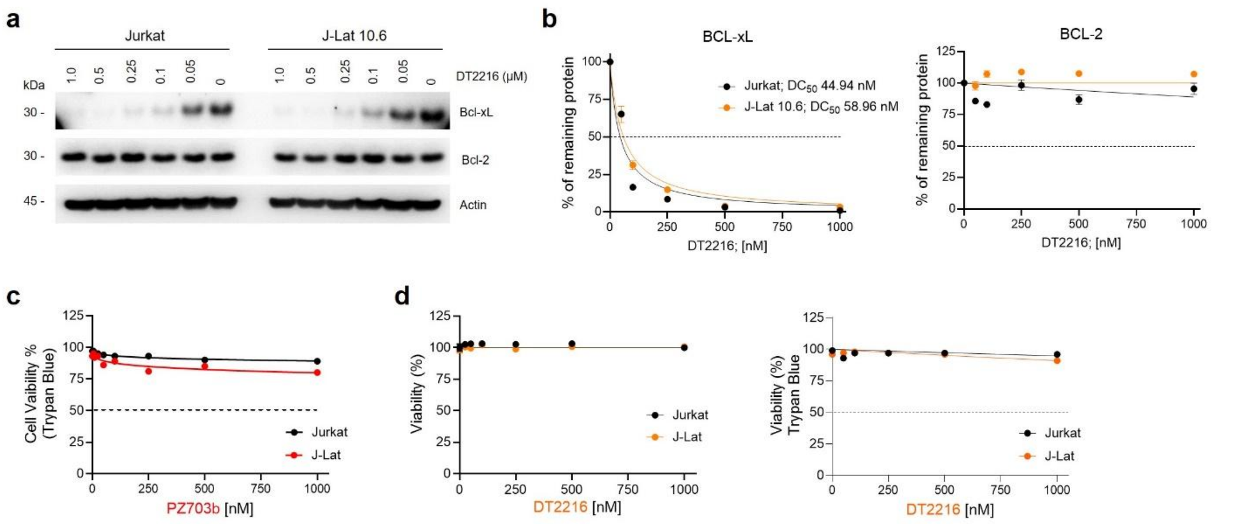

Supplementary Figure S3. DT-2216, a Bcl-xL PROTAC, selectively induces Bcl-xL degradation without cytotoxicity in Jurkat and J-Lat cells. (A) (B) DT-2216 efficiently degrades Bcl-xL but not Bcl-2 proteins in Jurkat and J-Lat 10.6 cell lines after 24 hours of treatment with increasing concentrations. DC50 represents the drug concentration required for 50% protein degradation. Actin was used as a loading control in immunoblot analyses. The density of untreated samples was set to 100% for protein levels; treated samples were first referenced to Actin and then normalized to untreated to calculate the reduction percentage. **(C) (D)** MTS and trypan blue exclusion assays were performed in Jurkat and J-Lat cells treated with increasing concentrations of PZ703b (**C**) or DT-2216 (**D**) for 24 hours.

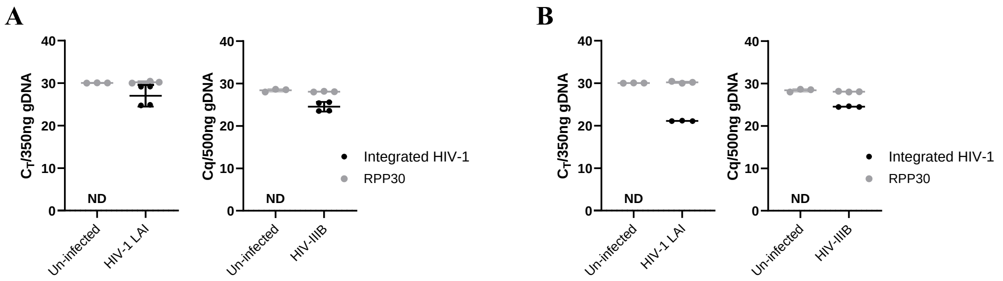

Supplementary Figure S4. Confirmation of HIV-1 Latency in the T_{CM} model of latency with ARTs. **(A)** RAL and ENF, or **(B)** RAL and DRV. Integrated HIV-1 DNA displayed average Ct values of 20 and 25, corresponding to approximately 300 to 3,000 copies of HIV-1 latent proviruses in the HIV-LAI and HIV-IIIB latently infected T_{CM} cells, respectively.

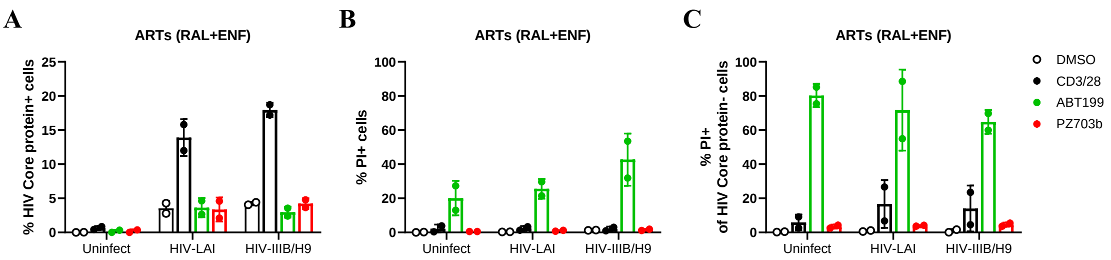

Supplementary Figure S5. The latency reversal and killing activities of PZ703b and ABT199 in the presence of a combination of integrase inhibitor and fusion inhibitor in T_{CM} model of latency. Naive CD4+ T cells were isolated from the peripheral blood mononuclear cells (PBMCs) of two healthy donors and established in a latent state by infection with HIV-LAI or HIV-IIIB/H9, or left as uninfected controls, as described in the Methods section.

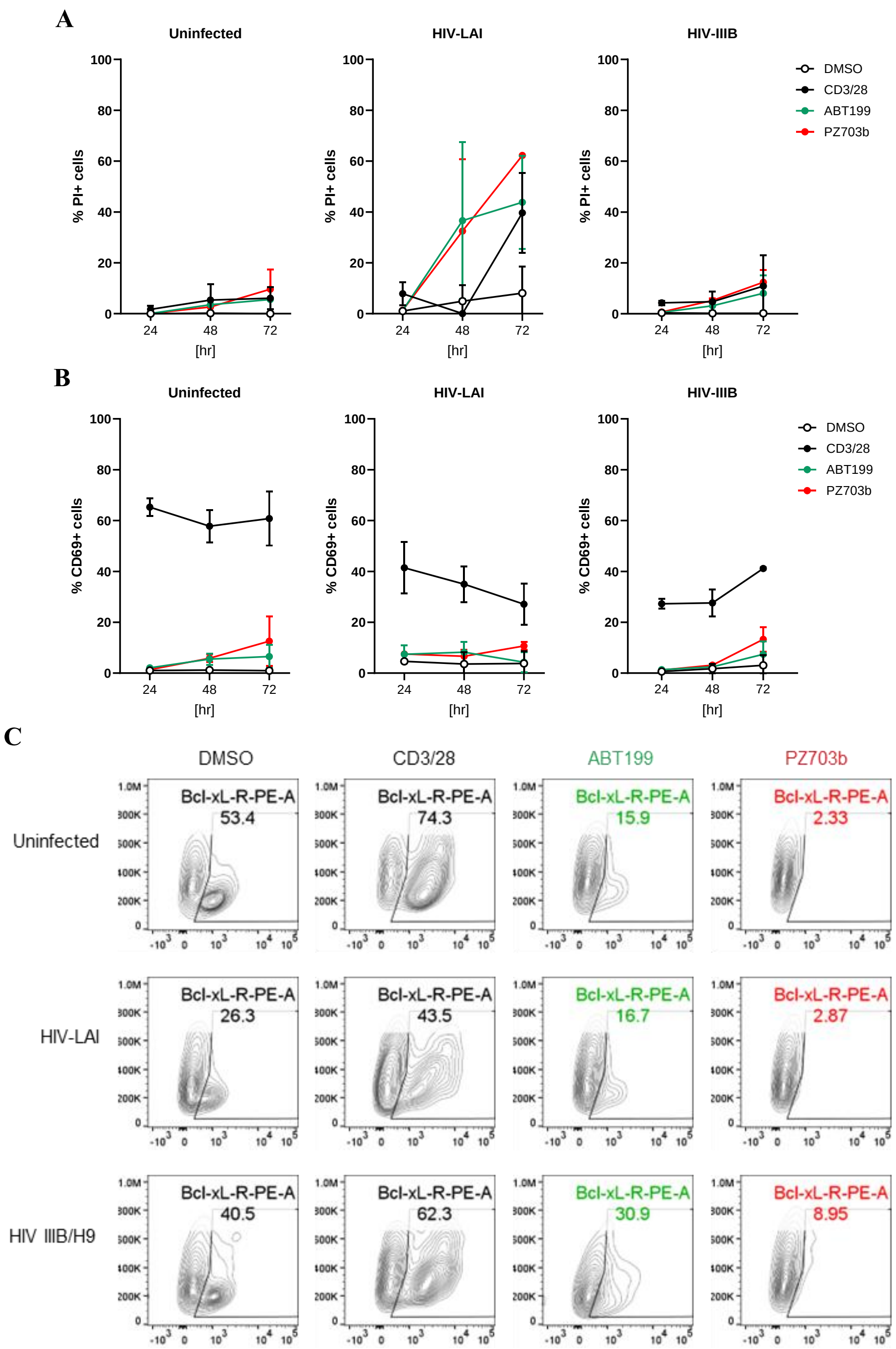

Supplementary Figure S6. Effects of PZ703b and ABT199 on Cell Toxicity, Global Activation, and BCL-xL Degradation in the T_{CM} model of latency. The frequency of propidium iodide-positive (PI+) cells as in **(A)**, CD69-positive (CD69+) cells as in **(B)**, and BCL-xL expression levels as in **(C)** following treatment of latently infected cells with DMSO, anti-CD3/CD28 antibody-conjugated beads, ABT199 (1 µM), or PZ703b (0.5 µM) for 24, 48, and 72 hours is presented as mean percentages ± SD from two biological replicates.

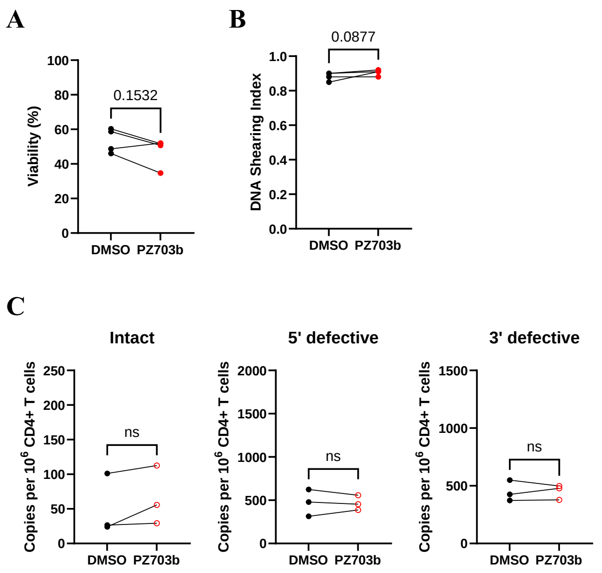

Supplementary Figure S7. shows **(A) (B)** the cell viability and shearing index of ex vivo CD4+ T cells and their corresponding DNA samples. **(C)** results of the IPDA assay of ex vivo reservoirs isolated from HIV patients without HCV co-infection.
